# Effect of 1-Ethyl-3-methylimidazolium Tetrafluoroborate and Acetate Ionic Liquids on Stability and Amyloid Aggregation of Lysozyme

**DOI:** 10.3390/ijms23020783

**Published:** 2022-01-11

**Authors:** Diana Fedunova, Andrea Antosova, Jozef Marek, Vladimir Vanik, Erna Demjen, Zuzana Bednarikova, Zuzana Gazova

**Affiliations:** Institute of Experimental Physics, Slovak Academy of Sciences, 040 01 Kosice, Slovakia; antosova@saske.sk (A.A.); marek@saske.sk (J.M.); vanik@saske.sk (V.V.); demjen@saske.sk (E.D.); bednarikova@saske.sk (Z.B.)

**Keywords:** amyloid aggregation, ionic liquids, protein stability, amyloid fibril polymorphism

## Abstract

Amyloid fibrils draw attention as potential novel biomaterials due to their high stability, strength, elasticity or resistance against degradation. Therefore, the controlled and fast fibrillization process is of great interest, which raises the demand for effective tools capable of regulating amyloid fibrillization. Ionic liquids (ILs) were identified as effective modulators of amyloid aggregation. The present work is focused on the study of the effect of 1-ethyl-3-methyl imidazolium-based ILs with kosmotropic anion acetate (EMIM-ac) and chaotropic cation tetrafluoroborate (EMIM-BF_4_) on the kinetics of lysozyme amyloid aggregation and morphology of formed fibrils using fluorescence and CD spectroscopy, differential scanning calorimetry, AFM with statistical image analysis and docking calculations. We have found that both ILs decrease the thermal stability of lysozyme and significantly accelerate amyloid fibrillization in a dose-dependent manner at concentrations of 0.5%, 1% and 5% (*v*/*v*) in conditions and time-frames when no fibrils are formed in ILs-free solvent. The effect of EMIM-BF_4_ is more prominent than EMIM-ac due to the different specific interactions of the anionic part with the protein surface. Although both ILs induced formation of amyloid fibrils with typical needle-like morphology, a higher variability of fibril morphology consisting of a different number of intertwining protofilaments was identified for EMIM-BF_4._

## 1. Introduction

Several dozen proteins and peptides have been found to form amyloid fibrils, whether or not associated with amyloid-related diseases, such as Alzheimer’s, Parkinson’s, Huntington’s, type II diabetes or hereditary systemic amyloidosis [1,2,3,4]. Amyloid fibrils have also been recently tested as novel biomaterials due to their specific properties such as high stability, strength, elasticity or resistance against degradation [5,6,7]. Amyloidogenic proteins have different chemical natures, no similarity in size, function or primary amino acid sequences. However, in their amyloid form, they display common properties such as the cross-β-sheet structure of the amyloid fibril core, in which continuous β-sheets are formed with the β-strands perpendicular to the long axis of the fibrils [8]. Fibrils with different morphology can arise depending on the conditions that induce amyloid formation [9,10,11]. This polymorphism is probably caused by the existence of various independent and competing pathways in the self-assembly process [12,13,14,15,16]. 

The nature of the solvent used for protein fibrillization in vitro represents one of the crucial conditions affecting the rate of fibrillization as well as the morphology of amyloid fibrils. Ionic liquids (ILs) have been introduced in many diverse areas of chemistry and industry as a “green” alternative to organic solvents [17,18,19,20]. ILs are organic salts consisting of ions, with a melting point below 100 °C. Therefore, many of them are liquid at room temperature. They have unique properties such as low volatility, high polarity [21,22], high ionic conductivity [23], high thermal stability or ability to dissolve different solutes [24]. The physico-chemical properties (density, viscosity, melting point, polarity, etc.) of ILs can be tuned by an appropriate combination of cations and anions to obtain solvent with desired properties for efficient utilization [17,20,23]. ILs have been identified as useful solvents for organic synthesis [25,26,27,28], in electrochemistry applications [29,30], as non-aqueous media for enzymatic reactions [31,32,33] or aqueous media for protein folding [22,34,35], and thermal stability studies [36,37,38,39]. Several studies have shown that ILs can effectively promote, alter or inhibit the process of protein amyloid aggregation. Two distinct classes of ILs, consisting of imidazolium- or ethylammonium-based cations and combined with a variety of inorganic or organic anions, have been studied in terms of their impact on the amyloid aggregation of different proteins, such as α-synuclein [40,41,42], Aβ-peptide variants [43,44], insulin [39,45], β-lactoglobulin [46] or albumin [47]. Byrne and Angel studied the effect of protic ionic liquids based on ethyl- and triethyl-ammonium cation on amyloid aggregation of lysozyme. The fibrils were resolvable in ethylammonium nitrate with 72% restoration of lysozyme enzymatic activity [48]. Kalhor et al. found that tetramethylguanidinium (TMG) ionic liquids combined with organic anions alter the kinetics of lysozyme fibrillization differently. Even though the ILs with TMG cation combined with tetrafluoroborate anion had no effect, ILs with acetate anion inhibited lysozyme fibrillization in the micromolar concentration range. The carboxyl group of the acetate anion has the main role in inhibition activity [49]. The inhibitory effect on lysozyme fibrils growth was also detected in the presence of 1-butyl-3-methylimidazolium bromide ionic liquid [50]. This IL was found to reduce β-sheet content in the secondary structure of the protein and suppress the exposure of hydrophobic Trp residues necessary for fibrillization. On the contrary, Silva et al. have reported the promotion of a lysozyme amyloid aggregation in the presence of ILs from two different families consisting of imidazolium or cholinium cation combined with different anions derived from organic acids. In contrast with previous findings, carboxylic acid functionality of acetate anion was responsible for inducing the formation of the protein β-structure content and fibrillization [51]. A brief review on the effects of various ILs on the amyloid aggregation of various proteins was published recently by Pillai and Benedetto [52]. They also emphasize that the same ILs may have a diverse or opposite effect on the aggregation of different proteins. Therefore, as they concluded, it is very hard to predict the effects of selected ILs on particular proteins in advance since the specificity of the physico-chemical interactions plays a crucial role here.

In this work, we have studied the kinetics of lysozyme amyloid formation and morphology of fibrils in the presence of 1-ethyl-3-methyl imidazolium (EMIM) ionic liquids with kosmotropic anion acetate (EMIM-ac) and chaotropic cation tetrafluoroborate (EMIM-BF_4_). We have selected these ILs due to their different kosmotropicity and mentioned contradictory effect of used anions on the stability and aggregation of proteins. We have found that despite different kosmotropicity, both ILs decrease the thermal stability of lysozyme and significantly accelerate lysozyme fibrillization in a dose-dependent manner, with a more prominent effect of EMIM-BF_4_. Using a special modified algorithm for the image and statistical analysis [53,54] and by correlation of obtained data with the hierarchical assembly model of fibrils [55,56], we have found that EMIM-BF4 induces the formation of mature fibrils with different content of intertwining protofilaments and protofibrils than EMIM-ac.

## 2. Results 

### 2.1. Kinetics of Lysozyme Amyloid Fibrillization in ILs

**Figure 1:**
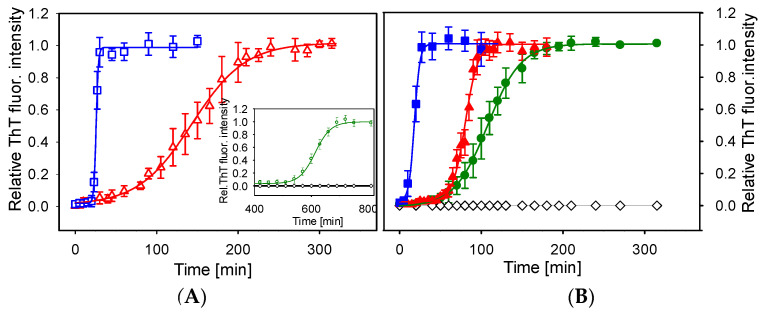
Kinetics of lysozyme fibrillization in the absence and presence of an increasing (*v*/*v*) concentrations of ILs. (**A**) EMIM-ac: 1%—red open triangles; 5%—blue open squares. Inset: EMIM-ac: 0%—black open diamonds; 0.5%—green open circles. (**B**) EMIM-BF_4_: 0%—black open diamonds; 0.5%—green circles; 1%—red triangles; 5%—blue squares. Lysozyme concentration was 2 mg/mL.

The effect of EMIM-BF_4_ and EMIM-ac on the kinetics of lysozyme amyloid fibrillization was studied by Thioflavin T (ThT) fluorescence assay. The restriction of free rotations of benzylamine and benzothiazole rings of ThT upon binding with amyloid fibrils leads to the increase of its fluorescence signal that is used to quantify the amount of fibrils. The data obtained for lysozyme fibrils formation in the absence and the presence of ILs at concentrations of 0.5, 1 and 5% (*v*/*v*) are presented in Figure 1. In the absence of ILs, no lysozyme fibrils were observed (Figure 1—black diamonds) at a prolonged time up to 13 h. The addition of ILs significantly promotes lysozyme fibrillization; the formation of amyloid fibrils was observed for all studied concentrations. 

To quantify observed differences, the kinetic parameters as lag-time (*t_lag_*), aggregation half-time (*t_half_*) and polymerization rate constant (*k_agg_*) were determined from kinetic curves fitted by the Boltzmann sigmoidal equation (see Section 4.3). The *t_lag_* corresponds to the time necessary for nuclei formation. The polymerization rate constant *k_agg_*, and the half-time of aggregation *t_half_* describe the growth phase during which the polymerization of oligomers into fibrils occurs. The obtained parameters are summarized in Table 1.

The most profound difference was the lag phase duration and the elongation phase steepness. The addition of 0.5% (*v*/*v*) of EMIM-ac and EMIM-BF_4_ reduced the time necessary for lysozyme fibrillization. The short fibrillation half-times observed mainly upon the addition of 1% or 5% (*v*/*v*) of ILs demonstrate the ability of ILs to considerably reduce the time needed for the formation of mature lysozyme fibrils. The decrease of the lag time *t_lag_* and half time of aggregation *t_half_* and the increase of *k_agg_* with increasing concentrations of ILs indicate that both ILs promote the lysozyme fibrillization not only by favoring the creation of nuclei but also by accelerating the elongation of fibrils. The effect of EMIM-BF_4_ was more prominent than the effect of EMIM-ac.

### 2.2. Thermal Stability of Lysozyme in the Presence of ILs 

**Figure 2:**
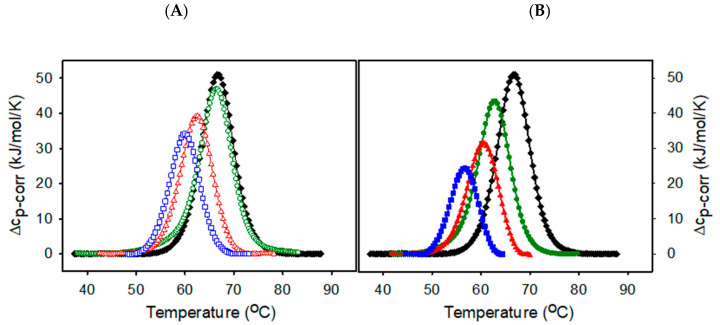
DSC thermograms of lysozyme at the absence (black) and at the presence of (**A**) EMIM-ac (0.5%—green open circles; 1%—red open triangles; 5%—blue open squares. (**B**) EMIM-BF_4_: 0.5%—green circles; 1%—red triangles; 5%—blue squares. Lysozyme concentration 2mg/mL, heating rate 1.5 °C/min.

It is generally accepted that amyloid aggregation of globular proteins results from a partially unfolded conformational state. Therefore, we have focused on studying the effect of ILs on the thermal unfolding of lysozyme by the differential scanning calorimetry (DSC). Figure 2 shows DSC scans of lysozyme in the absence and the presence of ILs at concentrations varying from 0.5% to 5% (*v*/*v*). The thermal denaturation of lysozyme in the absence of ILs is a reversible two-state process as follows from the ratio of van’t Hoff and calorimetric enthalpies being ~1 (ΔH_cal_ = 432.2 ± 1.5 kJ/mol, ΔH_vH_ = 448.5 ± 1.9 kJ/mol) with transition temperature T_d_ = 66.57± 0.02 °C. The ratio of van’t Hoff and calorimetric enthalpies ΔH_vH_/ΔH_cal_ is known to be a measure of the cooperative unit. 

The thermal stability of lysozyme decreases dose-dependently in the presence of studied ILs. With increasing EMIM-BF_4_ concentration the transition temperatures T_d_ are shifted to lower values, (Figure 2B), and calorimetric enthalpy change ΔH_cal_ decreases, indicating the destabilizing effect on the native lysozyme structure. The thermal denaturation is still highly reversible. The higher value of ΔH_vH_/ΔH_cal_ ratio, greater than 1, observed mainly for the highest ILs concentrations suggests the increase of cooperative unit favoring protein-protein interactions. Similar behavior is observed for EMIM-ac (Figure 2A), but the effect is less prominent. EMIM-BF_4_ is a slightly more effective destabilizer than EMIM-ac. Regardless of different kosmotropicity of acetate and tetrafluoroborate anions, when combined with chaotropic cation EMIM, both ILs destabilize lysozyme at acidic pH conditions within the lower to moderate concentration range. The thermal denaturation parameters of lysozyme in the absence and presence of ILs are summarized in Table 2.

The thermal unfolding of globular proteins proceeds through the breaking of van der Waals contacts, salt bridges and hydrogen bonds, followed by hydration of newly exposed hydrophobic core. The role of ions in altering the protein stability lies in modifying the protein structure strength or changing the free energy associated with hydration of the newly exposed core [57]. One possible mechanism of modulating protein stability by ions is a long-distance non-specific electrostatic screening of positively charged protein groups by anions. The lysozyme at pH 2.5 has a high positive net charge of about +18 [58]. Therefore, the neutralization of charges might affect the peptide backbone and thus alter the stability. From the studies of the effect of salts on lysozyme stability, Bye et al. suggested that, at low concentrations of anions, short-distance local electrostatic interactions between negatively charged anions and positively charged residues might also play a role and the effect, stabilizing or destabilizing, is protein- and ion-specific [57]. 

### 2.3. Morphology of Lysozyme Amyloid Aggregates

**Figure 3:**
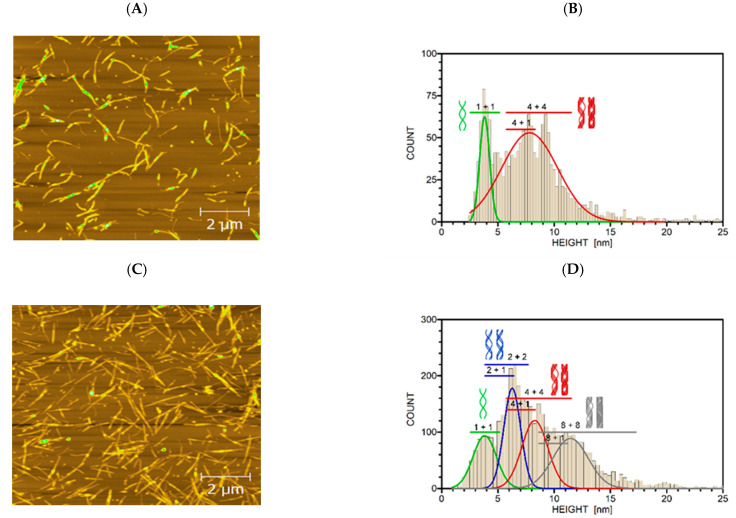
AFM image of lysozyme fibrils formed in 1% EMIM-ac (**A**) and EMIM-BF_4_ (**C**). The corresponding histograms of height distribution of fibril ridges with the schematic model of fibril types. (**B**) About 1600 fibril cross-section profiles were used to build histograms and (**D**) about 3900 fibril cross-section profiles were used to build histograms.

Further, we have studied the morphology of fibrils formed in 1% ILs by AFM and image analysis. At 0.5% and 5% ILs concentrations, the AFM images were not eligible for selected image analysis method due either to the too low amount of fibrils or a net of overlapping fibrils, respectively. Both ILs induced formation of amyloid fibrils with typical needle-like morphology. Figure 3A shows an AFM image of mature lysozyme fibrils obtained in the presence of 1% EMIM-ac after 4 h of incubation in fibrillization-inducing conditions, which corresponds to the plateau (equilibrium) phase of fibrillization kinetics. Figure 3C shows an AFM image of mature lysozyme fibrils formed in the presence of 1% EMIM-BF_4_, after 2 h of incubation in fibrillization-inducing conditions, which corresponds to the same position in the plateau (equilibrium) phase of fibrillization as for EMIM-ac. 

For more detailed characterization of fibril morphology, we used an image processing algorithm described in [53,54], modified for our purposes, to extract the distribution of fibril heights. Mean height values were statistically processed and compared with the hierarchical assembly model of fibrils [55,56]. The main advantage of this approach is the ability to extract the height values along the fibril ridges taking into account the intertwining of particular fibrils. According to the used model of fibril height [55,56], the protofilaments elongate due to the addition of monomeric partially folded intermediates. Then, protofilaments interact and intertwine to form protofibrils. Finally, protofibrils intertwine to fibrils. The obtained height histograms correspond to the distributions of fibril ridge heights. The most probable groups of fibril sizes are determined by the normal distribution fits of the histogram, as is seen in Figure 3B,D. The height histograms corresponding to different fibrils are partly overlapping due to the variability in the combination of the fibrils’ hierarchical distributions. This overlap broadens the height distribution, and therefore the fitted peaks represent fibril groups of similar parameters rather than individual fibril types. The actual distributions are furthermore influenced by fluctuations in fibril structures, background distortion and particularly the precision of the AFM device. 

In the presence of 1% EMIM-ac (Figure 3B), we observed two distinct populations of fibrils. A more noticeable population with a mean height of 3.7 nm corresponds to two protofilaments intertwining into protofibril (1 + 1). The second peak includes a broad population of fibrils consisting of two fibril types (4 + 1, 4 + 4). Populations of mature fibrils’ heights occur mainly in the interval of 4–12 nm. In the presence of 1% EMIM-BF_4_ (Figure 3D), the spectrum of fibril sizes is broader. The filament ridge heights extend to 15 nm. We have observed four prominent distribution peaks corresponding to the population of two protofilaments assembling into protofibril (1 + 1) and two protofibrils intertwining into mature fibrils (2 + 2) and their various combinations (2 + 1, 4 + 1, 4 + 4, 8 + 1, 8 + 8). The number of intertwining protofilaments is depicted above each histogram.

In the absence of ILs, lysozyme at 2 mg/mL concentration does not form amyloid fibrils even after 50 h of incubation. At concentrations 10 times higher (20 mg/mL) and the same fibrillization-inducing conditions, lysozyme formed only three fibril types (1 + 1, 2 + 1, 2 + 2) (Figure S1B).

The smallest fibril type is a flexible protofilament with a mean height of 2.6 nm ± 0.4 nm, near the lysozyme monomer particle mean size [56]. The second (3.7 ± 0.8 nm) peak corresponds to two protofilaments intertwining to protofibril (1 + 1). The third (6.1 ± 1.1 nm) peak of fibril types contains certain combinations of protofilament and protofibril (2 + 1, 2 + 2). There are practically no fibrils higher than 6 nm. The example of intertwining protofilaments (1 + 1) is illustrated in Figure S2. 

In the absence of ILs, there are primarily protofilaments, protofibrils (1 + 1) and a fraction of two intertwining protofibrils (2 + 1, 2 + 2). ILs-induced fibrils populations consist of a significant fraction of higher-order fibril types (4 + 1, 4 + 4, 8 + 1, 8 + 8). However, the fibril ridge profiles are smooth and irregular with non-measurable pitch distance. The regions of the obtained heights are in a broader range than data previously shown for lysozyme fibrils (mostly 2–8 nm) [51,59], indicating the ability of both ILs, and EMI-BF_4_ in particular, to induce the formation of morphologically distinct fibrils. Higher fibril heights up to 30 nm are reported for mixtures of laterally stacked or overlapped fibrils, not allowing valuable comparison [60,61].

### 2.4. Lysozyme Fibrils Secondary Structure

**Figure 4:**
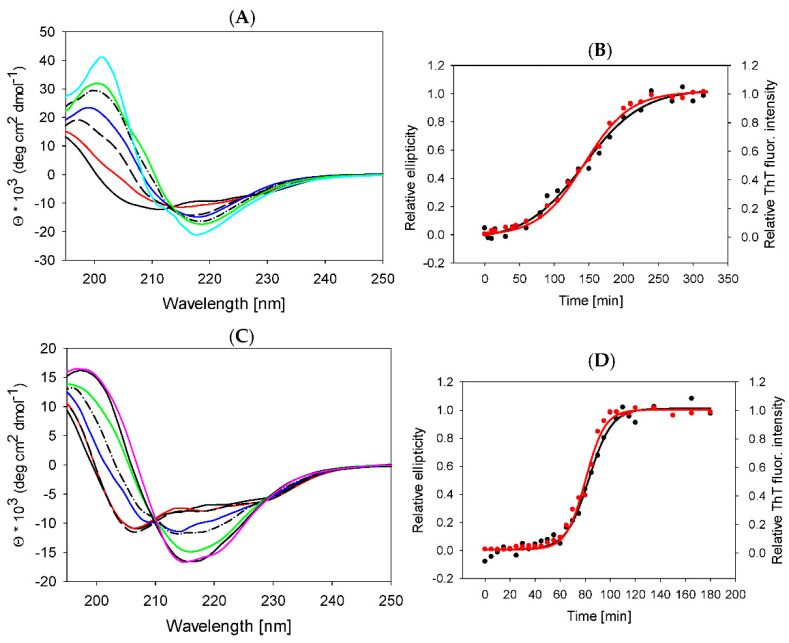
Far UV−CD spectra of lysozyme in 1% EMIM-ac (**A**) and 1% EMIM-BF_4_ (**C**). The samples were withdrawn at different time points of fibrillization (identical time point as used for the ThtT kinetics measurement). The arrows indicate the order of spectra with increasing time. A reduced number of spectra is shown for better readability. (**B**,**D**) Time dependence of the normalized value of mean residue ellipticity at 218 nm for all measured spectra—black circles, ThT fluorescence intensity—red circles (taken from Figure 1A).

To understand the changes in lysozyme secondary structure upon fibrillization and their potential impact on fibril morphology, we monitored the kinetics of lysozyme assembly by far-UV CD spectroscopy. Figure 4A,C show the CD spectra of lysozyme in the presence of 1% EMIM-ac and 1% EMIM-BF_4_ upon prolonged exposure to fibrillization-inducing conditions at increasing time points. Prior to fibrillization (Figure 4A,C—solid black line), the spectra display negative bands at 222 nm and 206 nm, typical for predominantly α-helical content. During fibrillization, lysozyme undergoes a transition from α-helical to β-sheet structures as an increasing peak of negative ellipticity at 218 nm and positive ellipticity at 200 nm appears with increasing time.

The spectra of mature fibrils formed in the presence of 1% EMIM-ac (Figure 4A—solid cyan line, t = 330 min) and 1% EMIM-BF4 (Figure 4C—pink line, t = 180) reveal the high content of β-sheets typical for amyloid fibrils. Since the peak at 218 nm is a marker of β-sheet structure, inevitably present in amyloid fibrils, we have used it to quantify fibrillization kinetics. A sigmoidal curve was obtained by plotting the normalized ellipticity value at 218 nm against time, pointing out the three-phase kinetics of fibrillization (lag phase, elongation phase and equilibrium phase) (Figure 4B,D—black circles). During the lag phase, nuclei are formed from partially unfolded monomers with low β-sheet content. With the formation of the β-sheet-rich structures during the elongation phase, the ellipticity at 218 nm increases. In the final plateau phase, corresponding to the maturation of fibrils, no additional changes in β-sheet content occur. The obtained CD kinetics mirror the ThT kinetics (Figure 4B,D—red circles). CD spectroscopy detects subtle changes in intra- and intermolecular secondary structure rearrangements, while the ThT assay is sensitive to the presence of intermolecular β-sheet content. The similar kinetic profiles obtained by CD and ThT measurements suggest that conformational rearrangement of lysozyme molecules from α- to β-rich structure is associated with the formation of irreversible pre-fibrillar species and protofibrils.

The particular secondary structure motifs were estimated from the spectra using the “Dichroweb” tool for semi-quantitative analysis of the structural composition of proteins [62]. The results are summarized in Table 3.

As follows from the analysis, both ILs slightly changes the secondary structure content of native lysozyme at room temperature. The α-helical content is reduced from ~34% to ~31% in both ILs, and β-sheets content increases from ~16% to ~19% in EMIM-ac and ~21% in EMIM-BF_4_ solutions. At the absence of ILs, lysozyme does not form amyloid fibrils. The amyloid fibrils prepared in 1% EMIM-ac in comparison with 1% EMIM-BF_4_ have a slightly higher content of β-sheet conformations (45% vs. 42%) and unordered structure (28% vs. 24%) on the expense of α-helix (5% vs. 9%) and β-turns (22% vs. 25%). These results show that the different morphology of fibrils formed in these ILs is mainly based on the effect of solvents on protofibril arrangement rather than differences in secondary structure content.

### 2.5. Molecular Docking

**Figure 5:**
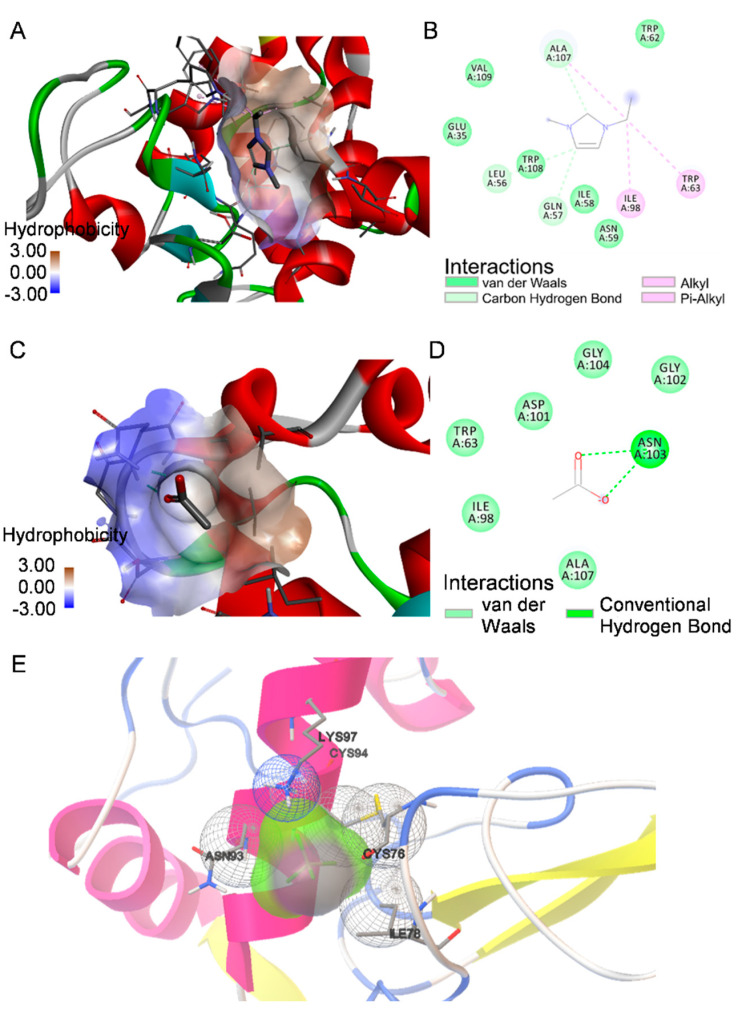
Hydrophobicity of amino acids residues on docking pose of lysozyme with (**A**) EMIM cation; (**C**) acetate anion. Molecular interaction diagrams of (**B**) EMIM cation and (**D**) acetate with amino acids residues of lysozyme. (**E**) Molecular interaction diagram of tetrafluoroborate anion (grey and green ball) to lysozyme from AutoDock analysis.

To shed more light on protein-ILs interactions, we performed docking calculation since the molecular insights into these interactions are sporadic, especially the destabilizing effects of ILs on proteins. Despite apparent limitations of docking calculations of protein interactions with solvent, they can provide insight to local interactions of cationic and anionic parts of ILs with protein and support experimental results. The lowest binding energy ranked complex of lysozyme with ILs with the most probable interactions is shown in Figure S3 with binding poses of all bound ions. The EMIM cation is located in the vicinity of the β-domain and C-helix (Figure 5A).

We have observed that the main interactions involved in binding of the EMIM cation to lysozyme are non-covalent carbon-hydrogen bonds with Gln57, Leu56 and Ala107, alkyl and pi-alkyl interactions with Trp63, Ile98 and Ala107 and van der Waals interactions with Ile58, Asn59, Trp62, Ala107, Trp108 and Val109 as depicted in Figure 5B. Singh et al. reported a similar binding mode of EMIM-ethylsulfate [63]. The acetate ion also binds to the β-loop and C-helix pocket, forming hydrogen bonds with Asn103 and van der Waals interactions with Trp63, Ile98, Asp101, Gly102, Gly104 and Ala107. The tetrafluoroborate anion forms van der Waals contacts with Ile78, Cys76, Cys94, Asn93 and Lys97. The estimated binding energy obtained from docking for EMIM, acetate and tetrafluoroborate is −3.7 kcal/mol, −2.85 kcal/mol and −0.38 kcal/mol. It is important to note that all ions bind in the same region of the β-domain loop and C-helix. Tetrafluoroborate anion binds weakly near the protein surface with lowest binding energy (−0.38 kcal/mol), suggesting that non-specific interactions with protein are more probable than specific local binding.

## 3. Discussion

The formation of amyloid fibrils is a complex process depending on the physico-chemical properties of protein as well as the solvent. Predicting the ILs effect on protein stability and aggregation is challenging due to the specificity of their interactions. Anions used in this study stand on the opposite side of the Hofmeister series, which ranks ions according to their effect on various biological phenomena such as protein solubility, stability or activity. The acetate is considered kosmotropic, while tetrafluoroborate is denoted as the chaotropic anion, which draws attention to their mutual ability to promote the aggregation kinetics of lysozyme. We have found that both ILs significantly accelerate the lysozyme fibrillization in a dose-dependent manner. The amyloid aggregation kinetics showed that the lysozyme amyloid formation follows nucleation growth (Figure 1). The rate-limiting step in nucleation-dependent amyloid fibrilization is the irreversible unfolding of the native structure followed by exposure of hydrophobic regions prone to form intramolecular interactions during the lag phase [64]. DSC measurements showed that despite the different kosmotropicity of used anions, both ILs at concentrations from 0.5% to 5% decreased the thermal stability of lysozyme. The transition temperature decreased with ILs concentration and is lower than the temperature of 65 °C set as amyloid-inducing factor except for 0.5% EMIM-ac (Figure 2). The lower transition temperature than working temperature probably ensures the exposure of hydrophobic protein core necessary for aggregation. The observed decrease of transition temperature with increasing ILs concentration follows a similar pattern as a decrease of lag-time or half-time of aggregation. 

Since the lysozyme is a highly charged basic protein, it is expected that long-range electrostatic interactions could be prominent in the modulation of protein stability along with other local/global ion-protein and bulk ion-solvent interactions. From docking calculations follows that the EMIM cation interacts locally with the hydrophobic cluster formed by the β-domain loop and C-helix. The binding of the EMIM cation interferes mainly with the region containing Trp62, Trp63 and Trp108. The disruption of the Trp62 native-like interactions in this domain was previously found to be responsible for the destabilization of the lysozyme [63,65]. Therefore, interactions of the EMIM cation with the lysozyme hydrophobic cluster are partially responsible for the destabilizing effect of both ILs. Furthermore, we assume that the specific interactions of ILs with protein are responsible for the different aggregation kinetics in acetate compared to tetrafluoroborate anion of ILs. The tetrafluoroborate anion shows only weak local interactions with the protein surface, suggesting that the non-specific long-range electrostatic screening of numerous positively charged groups on the protein surface is more likely to be exerted in the interactions. Moreover, the weak non-specific interactions of tetrafluoroborate as a low charge density and weakly hydrated chaotropic anion with apolar regions can reduce the Gibbs free energy associated with hydrating the newly exposed hydrophobic regions during unfolding, leading to destabilization. The DSC measurements support these suggestions. In the presence of EMIM-BF_4,_ a lower ΔH was found compared to the ILs-free conditions or EMIM-ac at given concentrations, showing that less energy is required for the protein thermal denaturation in the presence of EMIM-BF_4._ In the case of EMIM-ac, the acetate ion interacts locally with the protein β-domain loop and C-helix via hydrogen bonding and van der Waals interactions, which may be responsible for the slightly weaker effect of EMIM-ac in comparison with EMIM-BF_4_ on the thermal stability of lysozyme. The amphiphilic nature of the acetate anion may also allow long-range non-specific interactions with the non-polar groups on the protein surface, interfering with hydrophobic intermolecular contacts responsible for aggregation. Acetate is also known to dissociate insufficiently from the EMIM cation into isolated ions even in diluted solutions [66]. These properties might contribute to the milder effect of EMIM-ac on lysozyme stability compared to EMIM-BF_4_. 

We have also found that lysozyme fibril morphology can be modulated by the used ILs. The hierarchical architecture of the mature fibrils is based on the variability of intra- and inter-protofilament interactions. Whereas the main-chain interactions common to all polypeptides stabilize the constituent β-strands within the core of the fibril, the nature of the specific side-chains of the particular protein is responsible for the particular fibrillar scaffold by determining which regions of the sequence self-associate to form the intermolecular packing arrangement between the constituent β-sheets of the fibril core [67,68,69]. The fibril morphology analysis based on applying a hierarchical assembly model of fibrils provided detailed insight into the fibrils’ architecture. We distinguished several fibril types consisting of a various number of protofilaments and protofibrils intertwining into mature fibrils. In the presence of EMIM-BF_4_, we observed the most variable populations of fibrils (1 + 1, 2 + 1, 2 + 2, 4 + 4, 8 + 1, 8 + 8). In contrast, only three prominent populations of fibrils (1 + 1, 4 + 1, 4 + 4) were identified in the presence of EMIM-ac. The secondary structure of fibrils formed in different ILs varies only within the 3–4% range for particular secondary structure motifs. The fibrils prepared in 1% EMIM-ac have slightly higher content of β-sheet conformations and unordered structure and lower content of α-helix and β-turns than EMIM-BF_4_. We assume that EMIM-BF_4_ induces formation of fibrils with more complex architecture by affecting the protein hydration and facilitating the hydrophobic interactions.

The variability of ILs’ composition offers a great opportunity to tune the physico-chemical properties of ILs for targeted purposes. Many articles have reported the effect of ILs on protein stability and aggregation, identifying ILs capable of inhibiting/promoting fibril formation or destabilizing/stabilizing protein for long-term storage. Satish et al. studied the effect of EMIM, 1-butyl-3-methylimidazolium [BMIM], 1-octyl-3-methylimidazolium [OMIM] ILs combined with anions ethyl sulfate [ESO_4_] and chloride on the stability of lysozyme. They found that the presence of EMIM-based ILs increased, while BMIM and OMIM decreased, the stability of lysozyme in a dose-dependent manner following the trend EMIM-ESO_4_ > EMIM-Cl > BMIM-Cl > OMIM-Cl. The increased thermal stability was suggested to be caused by the interaction of the ILs cationic moiety with the negatively charged residues on the surface of lysozyme via electrostatic, hydrophobic forces/hydrogen bonding through amine protons with both cationic and anionic parts of ILs. The increase of ILs cation hydrophobicity facilitated interactions with the hydrophobic core of lysozyme and thus caused the disruption of the protein’s native structure [70]. Bisht at al. tested the effect of hydrophobic ILs with variable ammonium cations and fixed (trifluoromethylsulfonyl)imide [NTf2] anion. Even though the native structure of lysozyme in the presence of ILs was intact at room temperature, the protein thermal stability gradually decreased with an increase in the concentration of the ILs. This effect was attributed to the strong favorable hydrophobic interactions of the ILs with the amino acid residues of the protein [71]. The study of the effect of 1-Decyl-3-methylimidazolium chloride [DMIM][Cl], 1-Butyl-1-methylpyrrolidinium tetrafluoroborate [BMP][BF4] and 1-Butyl-1-methylpyrrolidinium bromide [BMP][Br] on the stability and activity of lysozyme revealed that imidazolium-based IL binds more strongly than pyrrolidinium-based ILs with lysozyme. BMP-BF_4_ and BMP-Br increased, while DMIM-Cl decreased, the stability of lysozyme. The docking results suggested that these ILs bind at the active site near the Trp108 in slightly different positions, mainly through electrostatic, hydrophobic and van der Waals interactions [72].

Kalhor et al. demonstrated the inhibitory effect of tetramethylguanidinium acetate on lysozyme amyloid aggregation leading to the formation of thinner fibrils, pointing out the importance of the carboxyl group presence for inhibition [49]. Basu et al. reported that ILs inhibited the lysozyme amyloid aggregation by suppressing the exposure of the hydrophobic clusters containing Trp residues prone to intramolecular interactions [50]. Byrne and Angell have shown that protic ILs based on ethyl-ammonium nitrate induced the unfolding and aggregation of lysozyme into fibrils and then promoted their dissolution to restore the native structure [48]. Interestingly, ethyl-ammonium nitrate ILs were reported to suppress the amyloid aggregation of insulin, emphasizing the importance of specific interactions of ILs with protein amino acids for inhibiting the amyloid aggregation [45]. Silva et al. studied the effect of imidazolium- and cholinium-based ILs combined with anions derived from organic acids. They have shown that all studied ILs promoted the lysozyme amyloid aggregation, inducing worm-like fibrils with 15–40 nm of width. They concluded that the presence of carboxylic acid functionality of acetate anion increases the ability of lysozyme to form β-sheet structures responsible for amyloid aggregation of lysozyme [51]. These results show that the effect of ILs on protein stability and aggregation strongly depends on protein structure and ILs composition, concentration and water content. A slight difference in specific interactions and the solvent properties may profoundly affect protein stability and aggregation. Our results are consistent with these findings, emphasizing the importance of specific interactions responsible for modulating amyloid aggregation kinetics and morphology. Therefore, the detailed study of the ILs effect on protein properties is of great interest.

In conclusion, this work revealed the ability of selected ILs to significantly accelerate the amyloid aggregation of lysozyme in conditions and time-frame when no fibrils are formed in the absence of ILs. Moreover, the used ILs were able to induce the formation of fibrils with different morphology. The application of ILs as modulators of protein amyloid aggregation is beneficial for further utilization of fibrils in biotechnological applications and understanding the molecular mechanism of protein amyloid aggregation and stability in complex solvents as ILs. 

## 4. Materials and Methods

### 4.1. Chemicals

Hen egg-white lysozyme (HEW lysozyme, E.C. number: 3.2.1.17, lyophilized powder, L 6876, 50,000 units mg^−1^ protein) and other chemicals (Thioflavin T, HCl) were purchased from Sigma-Aldrich Chemicals Company (St Louis, MO, USA). The ionic liquids (ILs), 1-ethyl-3-methylimidazolium tetrafluoroborate (>98%) (EMIM-BF_4_) and 1-ethyl-3-methylimidazolium acetate (95%) (EMIM-ac), were purchased from IoLiTec (Germany). For all measurements, lysozyme powder was dissolved in aqueous solution containing 0%, 0.5%, 1% and 5% (*v*/*v*) ILs to a final lysozyme concentration 2 mg/mL (140 μM). The pH was adjusted to 2.5 by a small amount of 1 M HCl before each experiment and carefully checked at every experimental step. The protein concentration was determined spectrophotometrically (UV/Vis JASCO V-630 spectrophotometer) using an extinction coefficient at 280 nm equal to 38,940 M^−1^ cm^−1^. Ultrapure deionized water (Milli-Q) was used for the experiments.

### 4.2. Preparation of Lysozyme Amyloid Fibrils

The lysozyme solutions (of 0%, 0.5%, 1% and 5% (*v*/*v*) ILs concentrations) were distributed to 2 mL tubes (sample volume 1.5 mL) and incubated at controlled temperature 65 °C in thermo-mixer with the constant agitation of 1200 rpm. The formation of lysozyme amyloid fibrils was examined by Thioflavin T fluorescence assay and confirmed by atomic force microscopy (AFM). 

### 4.3. Thioflavin T Fluorescence Assay

The lysozyme samples for the ThT assay were withdrawn from the 1.5 mL reaction mixtures at different time points and diluted to a final lysozyme concentration of 10 μM. The final concentration of ThT was 20 μM in all samples, and the samples were incubated at 37 °C for 45 min. Each experiment was performed in triplicate; the resulting data represent average values, and the error bars denote the average deviation. Measurements were performed in a 96-well plate by a Synergy MX (BioTek) spectrofluorimeter. The excitation wavelength was set at 440 nm, and the emission was recorded at 485 nm. The excitation and emission slits were adjusted to 9.0/9.0 nm, and the top probe vertical offset was 6 mm. The fluorescence intensities of the samples were normalized to the maximal fluorescence signal of mature amyloid aggregates taken as 100%. ThT fluorescence intensities were plotted as a function of time and fitted by a Boltzmann sigmoidal curve described by the following equation:

(1) $$y=y_{1}+\frac{y_{2}-y_{1}}{1+exp\left(-2\frac{t-t_{half}}{t_{half}-t_{lag}}\right)}$$

where *y* is the fluorescence intensity, *y*_1_ and *y*_2_ are the initial and final values of fluorescence intensity, $t_{half}$ denotes the fibrillization half-time (time at 50% of fluorescence maximum) and $t_{lag}$ corresponds to lag-time. The aggregation constant is calculated from equation $k_{agg}=2/\left(t_{half}-t_{lag}\right)$.

### 4.4. Differential Scanning Calorimetry (DSC)

DSC measurements were performed using a high-sensitivity VP-DSC microcalorimeter (MicroCal) at a heating rate of 1.5 °C/min and a temperature range of 25–110 °C. Measurements were carried out under a constant overpressure of 1.5 atm. All experimental curves were calibrated to the baseline obtained by heating the solvent in both cells. The calorimetric enthalpy change (Δ*H*_cal_) and van’t Hoff enthalpy change (Δ*H*_vH_) were determined by the non-linear model using Origin software supplied by MicroCal. The reversibility of the transition was assessed by the reproducibility of the calorimetric trace in the second heating cycle running immediately after cooling from the first scan. Excess heat capacity curves were plotted using Origin software supplied by Microcal.

### 4.5. Atomic Force Microscopy (AFM) 

The diluted lysozyme fibrils (10 µL) sample was placed on the surface of freshly cleaved mica and then left for 10 min to adsorb on the surface. After rinsing by the drop-wise addition of the ultrapure water, the samples were air-dried. AFM images were taken using a Veeco/Bruker Di Innova microscope in tapping mode in the air using an NCHV cantilever with a specific resistance of 0.01–0.025 Ω cm, antimony (n) doped Si with a typical resonance frequency 320 kHz, a radius of the tip curvature of 10 nm. The topographic images were corrected line by line for background trend effect with second-order polynomial fitting (Nanoscope, Burker). The AFM images were visualized by Gwyddion software [73].

### 4.6. Data Processing of AFM Topographies 

Data acquisition from AFM images: Ridge contours of fibrils were extracted semi-automatically using a modified live-wire algorithm [53,54]. Overlapping and crossing segments were excluded from the analysis. Lines perpendicular to fibril ridges were determined at each pixel, their parameters were estimated using the “median filtered differencing” algorithm [74]. Along each line, cross-sections were made through the surfaces of the fibrils. Height profiles along these lines were extracted and saved for further analysis. 

Fibril profiles processing: The cross-sections profiles from the previous step were projected into a plane perpendicular to the image plane and passed through the profile’s baseline. Using an automatic procedure, these profile projections were fitted with a 4-parameter Gaussian model (peak position, peak width, amplitude, and baseline). The goodness of fit expressed by the correlation coefficient is 0.95–0.98. Fibril ridge height values were then used to construct height distribution histograms.

Fibril heights histogram processing: The histograms of fibril ridge height values is a mixture of fibrils of different sizes. The multi-peak non-linear regression analysis was used to extract histograms of individual fibril types. This method comprises a model function consisting of a given number of normal distributions with maximal count, mean height, and standard deviation parameters. Fitting parameters were initialized using the visible parts of the distribution peaks. An optimization procedure with various peaks was performed to obtain the best fit. 

### 4.7. Circular Dichroism (CD) Spectroscopy

The CD measurements were performed using a JASCO J-810 spectropolarimeter in the far-UV region (190–250 nm) with a rectangular 1 mm path length cuvette with a scan rate of 50 nm min^−1^ at 25 °C. The samples withdrawn from the reaction mixture at different time points at different stages of fibrillization were diluted to a final lysozyme concentration of 10 μM. The dilution was also necessary for suppressing the contribution of the imidazolium cation to the spectra. Shown CD spectra represent the average of five consecutive scans. The secondary structure composition was evaluated using the CDPro software package Dichroweb [62]. CDSSTR and CONTIN methods with two reference database sets were used to estimate the percentage of secondary structure content. The normalized values of mean residue ellipticity at 218 nm were calculated from equation: *θ*_norm_ = (*θ* − *θ*_min_)/(*θ*_max_ − *θ*_min_), where *θ*_min_ corresponds to ellipticity at t = 0 min and *θ*_max_ to ellipticity at plateau phase.

### 4.8. Molecular Docking

The hen-egg white lysozyme monomer structure was taken from Protein Data Bank with PDB ID 193L at pH 4.3. Then, using the PDB2PQR server with Amber force field and PROPKA [75], the pH calculation was performed to decrease the pH to 2.5. The geometry of EMIM cation and BF_4_ and acetate anions were optimized at the DFT//B3LYP/6-31G level of theory using GAMESS suit [76] and Avogadro software. The docking input files were prepared by using AutoDockTools version 1.5.4. Docking studies were performed with AutoDock 4.2.6 [77] suite of programs. Since AutoDock does not provide parameters that recognize sp3 boron atoms, the parameter function was modified with added parameters suggested by Tiwari et al. [78]. The Autodock program was run to search for the lowest energy conformation within a volume space of a cube of 120 Å edge using 1.0 Å grid spacing to encompass the entire protein molecule. Docking to protein target was performed using Lamarckian Genetic Algorithm with an initial population size of 500, 2.5 × 10^6^ maximum number of energy evaluations, 27,000 maximum numbers of generations and a crossover rate of 0.8. The interactions of the docked structures with the lowest binding energy were analyzed using the Discovery Studio Visualizer, version 4.1. The binding pose of the BF_4_ anion was visualized by AutoDockTools, since Discovery studio does not support binding analysis of the sp^3^-hybridized Boron atom. 

## Figures and Tables

**Table 1:** Kinetic parameters derived from aggregation kinetics shown in Figure 1. *t_lag_* is the lag phase duration, *t_half_* corresponds to the aggregation half-time (the time of aggregation reaction at 50% ThT fluorescence intensity), *k_agg_* stands for aggregation constant, and R is the correlation coefficient.

	*t_lag_* (min)	*t_half_* (min)	*k_agg_* (min^−1^)	R
**EMIM-ac (% *v*/*v*)**				
0.5	558.60 ± 4.40	613.50 ± 2.20	0.036 ± 0.002	0.998
1	77.20 ± 4.10	143.10 ± 1.70	0.031 ± 0.003	0.996
5	19.80 ± 0.30	21.90 ± 0.20	0.925 ± 0.100	0.993
**EMIM-BF_4_ (% *v*/*v*)**				
0.5	67.00 ± 1.80	107.80 ± 1.40	0.049 ± 0.003	0.998
1	64.80 ± 1.50	80.30 ± 0.70	0.130 ± 0.010	0.997
5	11.30 ± 1.90	18.10 ± 0.80	0.290 ± 0.050	0.996

**Table 2:** Thermodynamics parameters derived from DSC thermograms depicted in Figure 2. R = reversibility (%).

	T_d_ (°C)	ΔHcal (kJ/mol)	ΔHvH (kJ/mol)	ΔH_cal_/ΔH_vH_	R (%)
Lys (2 mg/mL)	66.57 ± 0.02	432.20 ± 1.50	448.50 ± 1.90	1.04	98
**EMIM-ac (% *v*/*v*)**					
0.5	66.44 ± 0.02	399.90 ± 2.70	512.60 ± 3.20	1.28	95
1	62.56 ± 0.04	363.20 ± 2.50	445.90 ± 3.90	1.23	93
5	60.05 ± 0.04	270.10 ± 2.20	449.20 ± 6.40	1.66	72
**EMIM-BF_4_ (% *v*/*v*)**					
0.5	62.92 ± 0.03	362.20 ± 2.20	413.60 ± 3.20	1.14	96
1	60.38 ± 0.03	307.70 ± 3.00	412.90 ± 1.40	1.34	95
5	56.79 ± 0.03	177.40 ± 1.80	489.40 ± 3.90	2.42	89

**Table 3:** Secondary structure content estimated from CD spectra measured for 1% ILs concentration at the end of the incubation period. Open software Dichroweb was used for deconvolution of spectra.

	Native Lysozyme	Lysozymein ILs	1% EMIM-ac	1% EMIM-BF_4_
α-helix (%)	34	native	31	31
		fibrilar	5	9
β-sheet (%)	16	native	19	21
		fibrilar	45	42
β-turn (%)	21	native	21	18
		fibrilar	22	25
unordered (%)	29	native	29	30
		fibrilar	28	24

## Data Availability

Data will be available.

## Supplementary Materials

The following supporting information can be downloaded at: https://www.mdpi.com/article/10.3390/ijms23020783/s1.
