# Supplementary material for: Effect of 1-Ethyl-3-methylimidazolium Tetrafluoroborate and Acetate Ionic Liquids on Stability and Amyloid Aggregation of Lysozyme
Source: Int J Mol Sci. 2022 Jan 11;23(2):783. doi: 10.3390/ijms23020783 (PMC8775716; doi:10.3390/ijms23020783)
Supplement: Supplementary file 1 [file ijms-23-00783-s001.zip › ijms-1547274-supplementary.pdf]

Supplementary material

Effect of 1-ethyl-3-methylimidazolium tetrafluoroborate and acetate ionic liquids on stability and amyloid aggregation of lysozyme

Diana Fedunova*, Andrea Antosova, Jozef Marek, Vladimir Vanik, Erna Demjen, Zuzana Bednarikova, Zuzana Gazova*

[1] Institute of Experimental Physics, Slovak Academy of Sciences, Kosice, Slovakia

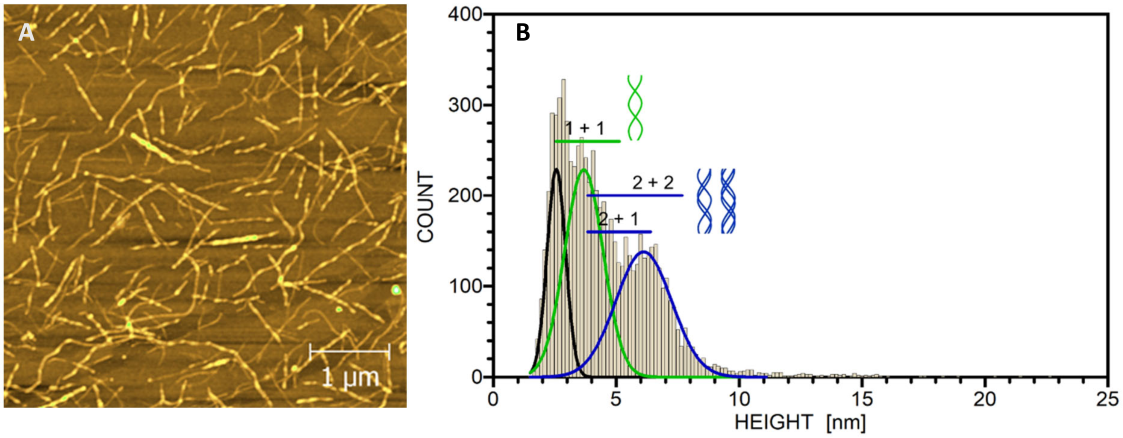

Figure S1. AFM image of lysozyme fibrils formed in the absence of ILs. (A) The corresponding histograms of height distribution of fibril ridges with the schematic model of fibril types. (B) About 7100 fibril cross-section profiles were used to build histograms

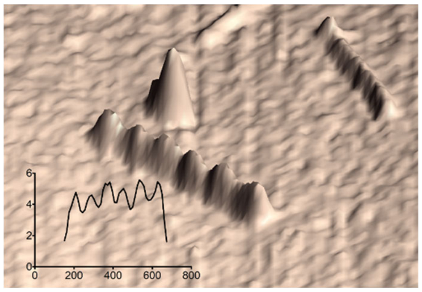

Figure S2. Example of intertwined (1+1) fibril type. Inset is the ridgeline height profile (pitch distance ~80 nm). 3D view was made using Gwyddion software.

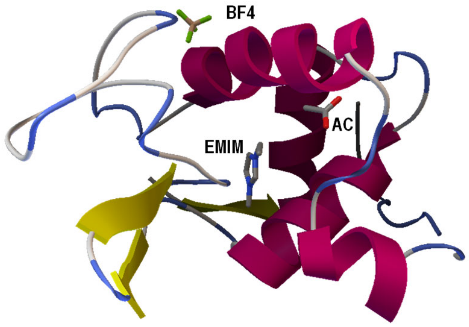

Figure S3. Accumulative image of binding poses of all studied ions. PDB ID (193L).
